# Role of self-control, financial attitude, depression, anxiety, and stress in predicting consumers’ online shopping addiction

**DOI:** 10.3389/fpubh.2024.1382910

**Published:** 2024-05-03

**Authors:** Yıldız Erzincanlı, Gönül Akbulut, Betül Buladi Çubukcu, Halil Gökhan Taş

**Affiliations:** ^1^Atatürk University, Aşkale Vocational High School, Erzurum, Türkiye; ^2^Atatürk University, Social Sciences Vocational High School, Erzurum, Türkiye

**Keywords:** online shopping addiction, financial attitude, depression, anxiety, stress

## Abstract

Online shopping addiction is a behavior that creates serious problems and has become increasingly prevalent in modern society. When addressing online shopping addiction, the direct or indirect causes of individuals’ shopping actions must be taken into consideration. The present study aims to examine the effects of self-control, financial attitude, depression, anxiety, and stress on online shopping addiction by determining online shopping addiction, self-control, and financial attitude levels of consumers. The sample of this study consists of 694 voluntarily participating consumers selected through convenience sampling methods from a city in Türkiye. Data were collected through Google Forms and uploaded to the SPSS 25.0 package program. During the research process, the relationship patterns between self-control, financial attitude, depression, anxiety, and stress on online shopping addiction were examined by using structural equation modeling. It was determined in this study that consumers have moderate levels of financial attitude and self-control, and low levels of online shopping addiction. Given the results related to the relationships and hypotheses between variables, anxiety, depression, and financial attitude were found to have statistically significant effects on online shopping addiction, whereas stress and self-control were found to not have a significant effect. Within the scope of this study, it was identified that anxiety and depression positively affect online shopping addiction, whereas financial attitude has a negative effect.

## 1 Introduction

With the rapid development of e-commerce in recent times, the usage rate of online shopping has also increased. According to the 2022 e-commerce bulletin in Türkiye, the e-commerce volume reached 800.7 billion TL with a 109% increase in comparison to the previous year. The number of orders in 2022 increased by 43% from the previous year, rising from 3.347 billion to 4.787 billion. Retail e-commerce volume in 2022 reached 458 billion TL (1, 2). As reported in the “Digital Türkiye February 2022” report prepared by the Global social media agency in collaboration with We Are Social and Hootsuite, the number of online shopping users in Türkiye increased by 3.6 million people in the last year, reaching 40.84 million, a 64% preference among internet users for purchasing goods or services through virtual stores (3). The emergence of online shopping opportunities with the advancements in internet-based data technologies led to a shift in consumption habits and the development of new types of addiction (4), with online shopping addiction being one of them.

Recent studies indicated that individuals spend approximately half of their waking hours struggling with desires and conflicting wishes (5). Even though not all desires that individuals experience are problematic, many can be problematic on an individual or even societal level. Managing problematic and conflicting desires requires constant effort from individuals. Success in navigating the constant flow of desires is associated with an individual’s ability to exercise self-control. Therefore, a variable in this study is self-control. The ability to regulate one’s emotions, thoughts, and behaviors is referred to as self-control. As stated by Baumeister (6), self-control is the capacity of an individual to alter their responses to the situation they find themselves in or to stimuli in their environment. Studies carried out on self-control showed that it is effective in every aspect of life (7). In the present context, online shopping addiction (OSA) is expressed as the inability of an individual to control their online shopping activities. According to Kraepelin (1909), shopping addiction arises from an individual’s lack of self-control. Individuals may decide to engage in shopping almost every day, and they often act unconsciously and cannot maintain self-control due to impulsive actions during the purchasing moment (8). Low self-control was associated with compulsive buying (9, 10) and behavioral addictions (11, 12). A study carried out by Rose and Dhandayudham (13) indicates that potential predictors of OSA include low self-esteem, low self-control, negative emotional state, depression, anxiety, and stress.

One of the variables in this study is depression-anxiety and stress. As stated in previous studies, factors such as entertainment, happiness, pleasure, boredom, depression, and stress are among the causes of online shopping addiction (14). Individuals are observed to engage in shopping, sometimes without the actual need, to alleviate stress, even occasionally without using the purchased products (15).

Depression, which is one of the most commonly observed mood disorders in individuals with online shopping addiction, is a condition that results in negative consequences both physically and emotionally after a prolonged period of sadness and unhappiness and has become increasingly prevalent recently (16). Anxiety is another frequent condition in individuals with online shopping addiction, typically characterized by fear and threat perception when facing a negative situation, often involving concerns about the future (17). Stress, defined as a continuous feeling of tension, arises when an individual does not feel sufficiently successful (16). Anxiety, depression, and stress are general emotional problems. During these problematic processes, individuals experience isolation, excessive restlessness, and tension, leading them to turn to online shopping, thereby increasing addiction tendencies (18). Many studies also indicated that online shopping is a method used to reduce tension in situations such as anxiety, depression, and stress (19).

Another variable examined in the present study is financial attitude. Considering Sen’s (20) capability theory, it is assumed that financial abilities can be a significant determinant in online purchasing. Financial attitudes in individuals’ lives affect their current and future behaviors (21). Financial attitude of an individual is a process that shapes emotions, thoughts, beliefs, and behaviors related to financial matters (22, 23). Individuals with poor financial attitude skills may make unhealthy decisions regarding online purchases, influencing their buying behavior. As reported in a study carried out by Ahmetoğulları (24), consumers’ financial attitudes affect their online shopping behavior.

Factors determining the novelty of this study include, firstly, the limited number of studies in the literature that concurrently investigate online shopping addiction with numerous factors, filling a gap in the literature on this subject. Secondly, due to the limited knowledge about the relationship between shopping addiction and its potential determinants, characterizing the patterns of online shopping addiction is challenging. The results of this study facilitate a better understanding of online shopping addiction and provide significant insights for practitioners.

Due to the limited number of studies in this field and since the present study aims to investigate which factors affect online shopping addiction, this study focuses on examining the effects of self-control, anxiety, depression, stress, and financial attitude variables on online shopping addiction. In this context, the final model was determined as “Consumers’ self-control, depression, anxiety, stress, and financial attitude significantly affect online shopping addiction.”

To achieve the objectives of the study, the following hypotheses were tested;

*H1*: DASS has a significant impact on online shopping addiction.

*H1a*: Anxiety has a significant effect on shopping addiction.

*H1b*: Depression has a significant effect on online shopping addiction.

*H1c*: Stress has a significant effect on online shopping addiction.

*H2*: Self-control has a significant effect on shopping addiction.

*H3*: Financial attitude has a significant effect on online shopping addiction.

## 2 Material and method

### 2.1 Data collection instruments

#### 2.1.1 Personal information form

The form includes 5 items related to the demographic and other personal characteristics of the consumers participating in this study. These questions were formulated by the researchers and they address the consumers’ genders, ages, marital statuses, and income levels, as well as the items related to the amounts spent on online purchases.

#### 2.1.2 Online shopping addiction scale

To measure consumers’ online shopping addiction, the Online Shopping Addiction Scale developed by Zhao et al. (25) and adapted into the Turkish language by Yılmaz et al. (26) was used in this study. The scale consists of five sub-dimensions: “Salience-Tolerance,” “Mood Change,” “Recurrence,” “Conflict,” and “Deprivation,” comprising 18 items in a 5-point Likert scale (26). In this study, the overall Cronbach’s Alpha value of the scale was found to be 0.958. The reliability levels of the overall scale and its sub-dimensions were determined to be high (Cronbach’s Alpha >0.70).

With established validity and reliability, this scale is a reliable and theory-based tool for measuring online shopping addiction. Moreover, the scale can be used by psychiatrists and psychologists working in the field of addiction for preliminary and final evaluations, as well as in cyberpsychology to examine the relationship between technology and human behaviors. The scale, consisting of 18 items, was preferred in the present study due to its effectiveness, as the response time is not long.

#### 2.1.3 Short self-control scale

The 13-item Short Self-Control Scale, developed by Tangney et al. (27) and adapted into Turkish by Nebioğlu et al. (28), was utilized to measure consumers’ self-control. The Short Self-Control Scale used in this study consists of a total of 13 items prepared in a 5-point Likert-type format with options ranging from “completely disagree” to “completely agree.” There are 4 positive and 9 negative items in the scale, and scoring is done in reverse for the negative items. The lowest score that can be obtained from the scale is 13, and the highest one is 65. A high score on the scale indicates a high level of self-control. The scale consists of 2 subscales: self-discipline and impulsivity. The overall Cronbach’s Alpha value for the scale in this study was determined to be 0.944. The reliability levels of the overall scale and its sub-dimensions were found to be high (Cronbach’s Alpha >0.70).

#### 2.1.4 Depression, anxiety, stress scale (DASS 21)

The Depression, Anxiety, Stress Scale (DASS 21) developed by Henry and Crawford (29) and Mahmoud et al. (30) was used to determine consumers’ levels of depression, anxiety, and stress. The adaptation of the scale into Turkish was carried out by Yılmaz et al. (16). The scale comprises three sub-dimensions: “Depression,” “Anxiety,” and “Stress,” with a total of 21 items in a 4-point Likert scale (16). In this scale, responses are coded as follows: 0 for “not suitable to me,” 1 for “partially suitable to me,” 2 for “mostly suitable to me,” and 3 for “completely suitable to me.” Subscale scores range from 0 to 28, with higher scores indicating higher levels of depression, anxiety, and stress. Since reliability levels were found to be higher than 0.70 for anxiety, depression, and stress measurements, it was determined that the reliability is at a high level (Cronbach’s Alpha; Anxiety: 0.90; Depression: 0.88; Stress: 0.85).

#### 2.1.5 Financial attitude scale

The Financial Attitude Scale developed by Onur and Nazik (21) was used to analyze consumers’ financial attitudes. This scale is unidimensional and consists of 24 items measured on a 5-point Likert scale. In this multiple-choice test with a statement format, each statement is rated on a scale of 1–5 and ranked as follows: “5 = Strongly Agree, 4 = Agree, 3 = Partially Agree, 2 = Disagree, and 1 = Strongly Disagree.” A single total score can be obtained from the scale, with the lowest score being 24 and the highest score being 120. A high score indicates a positive attitude toward individual finances. The reliability of the Financial Attitude Scale was calculated based on the alpha value using Cronbach’s Alpha coefficient, and the scale’s original internal consistency coefficient is 0.97. In this study, the overall Cronbach’s Alpha value for the scale was determined to be 0.979. Given that the reliability level of the scale exceeds 0.70, it was established that the reliability is at a high level.

### 2.2 Procedure

In parallel with the purpose, this study was designed using a relational survey model. Accordingly, participants were provided with necessary explanations regarding the purpose of the study, the anonymous utilization of the obtained data, and the voluntary participation principle in this study in order to collect data. The sample of the research consists of 694 volunteer consumers selected through convenience sampling, one of the non-probabilistic sampling methods, from a city in Turkey. The link (insert link) containing the data collection instruments was disseminated to the target population through national associations, unions, and civil society organizations. During this process, online forms were sent to members of these institutions via applications such as WhatsApp, and the data collection process was completed in approximately 3 months.”

### 2.3 Data analysis

This study was designed using the relational survey model. The data collection was performed online in a city in Türkiye in 2023 by using Google Forms. The analysis of the data obtained in the present study was conducted by making use of the LISREL 8.7 and SPSS 25.0 software packages. Significance levels were set at *p* = 0.05 and *p* = 0.01, and the reliabilities and validities of the scales used in the research were initially examined.

The reliability levels of the scales used in this study were calculated using the Internal Consistency method and the Cronbach Alpha reliability criterion. Confirmatory factor analyses were conducted to test the validity of the scales. Various fit indices were used to determine the adequacy of the model in confirmatory factor analysis. In addition to the Chi-square goodness-of-fit index, fit criteria such as IFI, CFI, RMSEA, GFI, and RMR were also considered in this study (31). Structural equation models were used to examine the results related to the research hypotheses. Normal distribution analyses and measures of central tendency were used to examine the distribution of data obtained in the research, and participants’ levels of participation in each measurement were examined using mean and standard deviation values. Frequency and percentage analyses were conducted to examine the participants’ demographic characteristics.

## 3 Results

### 3.1 Participants

The demographic characteristics of participants are shown in Table 1.

**Table 1:** Demographic characteristics of participants.

Variables	Group	*n*	%
Gender	Female	374	53.90
	Male	320	46.10
Age	18–28	167	24.10
	29–39	242	34.90
	40–50	211	30.40
	51–60	74	10.70
Marital status	Single	233	33.60
	Married	461	66.40
Income level	1,000–4,000 TL	118	17.00
	5,000–10,000 TL	162	23.30
	11,000–20,000 TL	321	46.30
	21,000–30,000 TL	63	9.10
	31,000 TL and higher	30	4.30
Monthly amount of online purchases	500–1,500 TL	492	70.90
	1,600–3,000 TL	128	18.40
	3,100–5,000 TL	29	4.20
	5,100–7,500 TL	21	3.00
	7,500 TL and higher	24	3.50
Total		694	100

In the context of this study, a total of 694 participants were contacted, with the majority being female (*n* = 374, 53.90%), aged between 29 and 39 years (*n* = 242, 34.90%), married (*n* = 461, 66.40%), having an income range of 11,000–20,000 (*n* = 321, 46.30%), and monthly online shopping expenditures ranging between 500 and 1,500 (*n* = 492, 70.90%).

### 3.2 Data distribution and descriptive results

In this section, findings related to the descriptive results and the distribution of data obtained in the present study are presented (Table 2).

**Table 2:** Data distribution.

Measurements	Central tendency		Kurtosis-Skewness	
	Mean	Median	Kurtosis	Skewness
Anxiety	7.54	5.50	−0.551	0.859
Depression	6.45	5.00	−0.665	0.646
Stress	7.22	6.00	−0.598	0.697
Financial attitude	79.72	94.00	−1.099	−0.859
Self-discipline	11.07	10.00	−1.377	0.128
Impulsivity	12.70	9.00	−1.581	0.388
Self-Control	23.77	19.00	−1.611	0.321
Significance – Tolerance	17.31	14.00	−1.546	0.326
Mood change	7.13	6.00	−0.682	0.819
Deprivation	6.36	5.00	0.121	1.152
Recurrence	6.55	5.00	−0.210	1.011
Conflict	6.25	5.00	0.107	1.122
Shopping addiction	43.61	37.00	0.158	1.172

Given the results of the normal distribution analysis, it was determined that the data obtained, due to the proximity of the measures of central tendency, namely mean and median, and the kurtosis and skewness being within ±2, originated from a normal distribution (32). Additionally, given the sufficient number of participants included in the study (*n* > =30), parametric methods, which are statistically more robust according to the central limit theorem, were employed (33) (Table 3).

**Table 3:** Descriptive results.

Measurements	Mean	SD
Anxiety	7.54	5.47
Depression	6.45	5.04
Stress	7.22	4.77
Financial attitude	79.72	26.00
Self-discipline	11.07	4.63
Impulsivity	12.70	6.14
Self-control	23.77	10.31
Significance – Tolerance	17.31	7.34
Mood change	7.13	3.52
Deprivation	6.36	3.39
Recurrence	6.55	3.39
Conflict	6.25	3.52
Shopping addiction	43.61	17.57

The scores obtained from the DASS scale, designed in the Likert format, were used to calculate the levels of participants’ responses with a point range of 15.75. In this case, the initial score of 0 points on the Likert scale is augmented by a 15.75-point interval, resulting in the calculation of intervals corresponding to each measurement level. Therefore, the range of 0–15.75 represents “Strongly Disagree,” 15.75–31.50 represents “Disagree Somewhat,” 31.50–47.25 represents “Agree Somewhat,” and 47.25–63 represents “Strongly Agree.” When examining the average and standard deviations of participants’ anxiety dimension (7.54 ± 5.47), depression dimension (6.45 ± 5.04), and stress dimension (7.22 ± 4.77), it was determined that they did not strongly agree with these dimensions.

For scales created in the Likert format, a point interval of 0.8 (4/5 = 0.80) was used to calculate the levels of participants’ responses based on the scores obtained. In this case, the Likert scale’s starting point of 1 point is augmented by a 0.8-point interval, resulting in the calculation of intervals corresponding to each measurement level. Thus, the range of 1–1.80 represents “very low,” 1.81–2.6 represents “low,” 2.61–3.4 represents “medium,” 3.41–4.2 represents “high,” and 4.21–5.0 represents “very high.” If the scale is calculated with the total score, then these intervals need to be multiplied by the number of items (34).

The participants’ level of financial attitude was determined to be moderate, with a mean of 79.72 ± 26.00.

Examining the participants’ self-control levels, it was found to be at a moderate level with a mean of 23.77 ± 10.31. Exploring the sub-dimensions of self-control, the sub-dimension of self-discipline was found to be at a moderate level with a mean value of 11.07 ± 4.63, whereas impulsivity was at a low level with a mean of 12.70 ± 6.14.

The participants’ level of online shopping addiction was identified as low, with a mean of 43.61 ± 17.57. Analyzing the sub-dimensions of shopping addiction, the dimension of clarity was found to be at a moderate level with a mean of 17.31 ± 7.34. The dimension of mood alteration was low with a mean of 7.13 ± 3.52, deprivation was low with a mean of 6.36 ± 3.39, recurrence was low with a mean of 6.55 ± 3.39, and conflict was low with a mean of 6.25 ± 3.52.

### 3.3 Relationships between variables and results for hypotheses

In this section, hypotheses established in accordance with the objective of this study were tested using Pearson’s correlation analysis.

As seen in Table 4, when examining the results of the correlation analysis, a statistically significant, strong, and negative relationship (*r* = −0.728; *p* < 0.01) was found between participants’ financial attitudes and the anxiety dimension, a sub-dimension of the Depression, Anxiety, and Stress (DASS) scale. This finding suggests that participants’ financial attitudes will decrease significantly as their anxiety dimensions increase. Moreover, there was a moderately significant and negative relationship between the levels of financial attitudes and the depression dimension (*r* = −0.410; *p* < 0.01), as well as the stress dimension (*r* = −0.446; *p* < 0.01).

**Table 4:** Analyses of the relationships between variables.

Variables		1	2	3	4	5	6
Anxiety	r	1	0.462*	0.542*	0.728*	0.521*	0.576*
Depression	r		1	0.607*	0.410*	0.307*	0.362*
Stress	r			1	0.446*	0.412*	0.400*
Financial attitude	r				1	0.508*	0.716*
Self-control	r					1	0.447*
Shopping addiction	r						1

*p < 0.01; Pearson’s correlation.

Furthermore, a positive and moderately significant relationship was found between participants’ self-control levels and the anxiety dimension (*r* = 0.521; *p* < 0.01), as well as the stress dimension (*r* = 0.412; *p* < 0.01), both being sub-dimensions of the DASS scale. This result suggests that participants’ self-control levels will also significantly improve when they improve their anxiety and stress dimensions. Additionally, a low-level positive relationship was determined between self-control levels and the depression sub-dimension (*r* = 0.307; *p* < 0.01).

Moreover, a moderately significant and negative relationship (*r* = −0.508; *p* < 0.01) was found between participants’ self-control levels and their levels of financial attitudes. This result suggests that, as participants increase their financial attitudes, their self-control levels will decrease moderately.

In terms of participants’ levels of online shopping addiction and the anxiety dimension (*r* = 0.576; *p* < 0.01), a positive moderately significant relationship was determined. This finding indicates that as participants improve their anxiety dimension, their levels of online shopping addiction will moderately improve. Additionally, a low-level positive relationship was identified between levels of online shopping addiction and the depression sub-dimension (*r* = 0.362; *p* < 0.01), as well as the stress dimension (r = 0.400; *p* < 0.01).

The level of online shopping addiction among participants was found to have a significantly high negative correlation with their financial attitude levels (*r* = −0.716; *p < 0.01). This result indicates that as participants enhance their financial attitudes, the levels of shopping addiction are expected to decrease significantly.

Additionally, a positively moderate significant relationship was identified between participants’ levels of online shopping addiction and their self-control levels (*r* = 0.447; *p < 0.01). This finding suggests that as participants improve their self-control levels, there is a moderate improvement expected in their levels of online shopping addiction.

### 3.4 Study model and hypotheses

**Figure 1:**
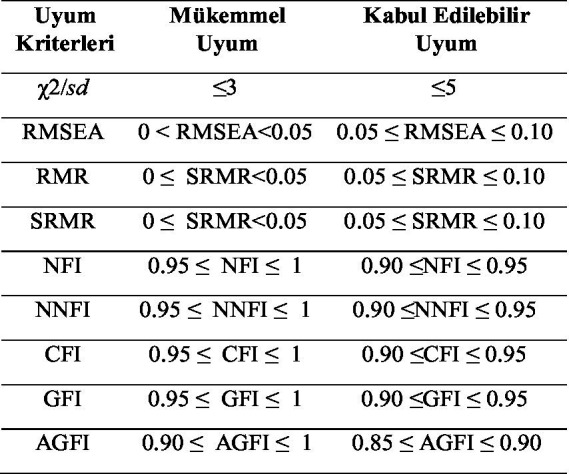
Fit criteria ranges used in this study. Source: Schermelleh-Engel and Moosbrugger (31).

In this section of the study, the results of the model, which was constructed in accordance with the objectives of the research and the hypotheses related to the model, are presented (Figure 1).

### 3.5 Results related to study hypotheses and model

**Figure 2:**
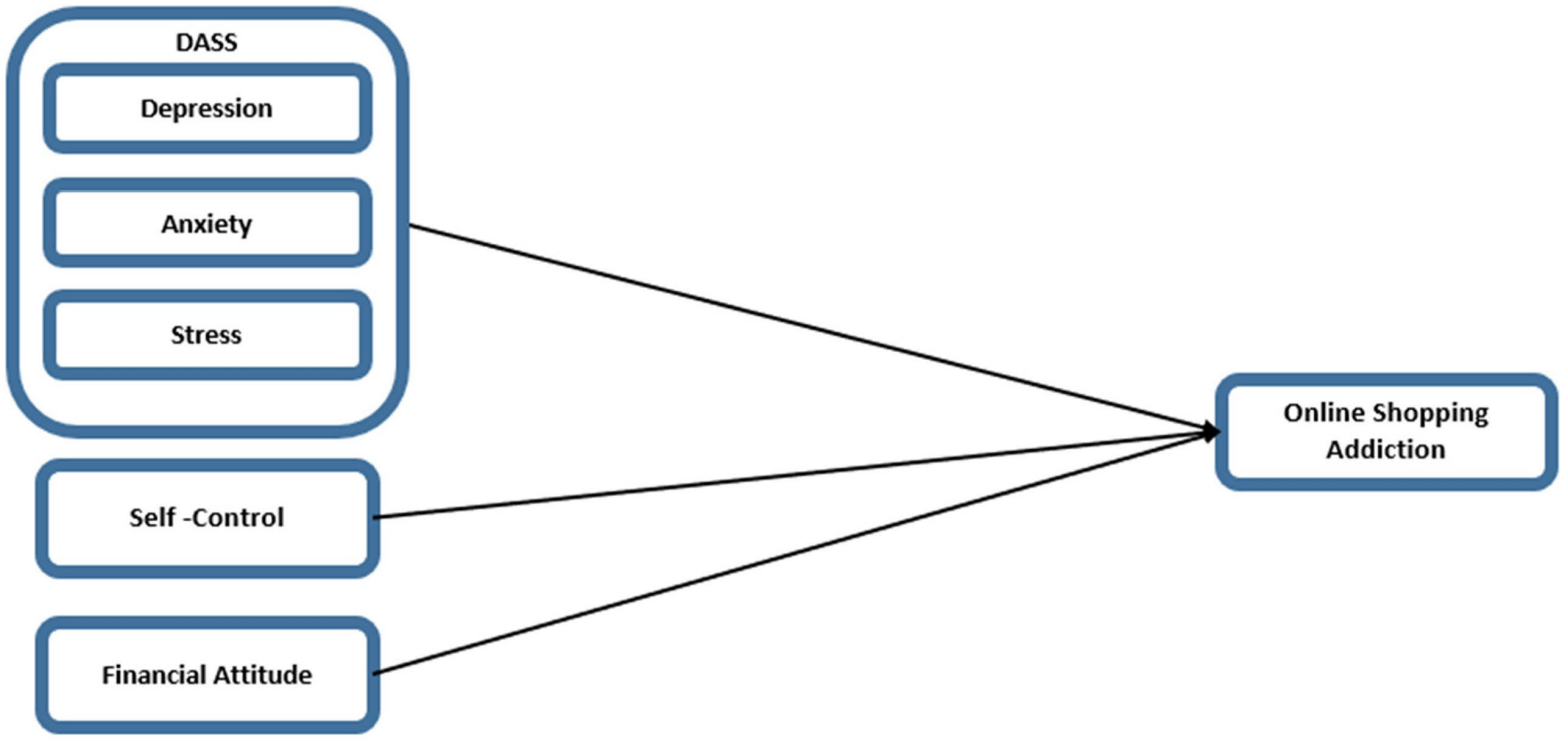
Study model.

Examining the study model and hypotheses, the path diagram of Structural Equation Modeling (SEM) is presented in Figure 2, with statistical values corresponding to the analysis results provided in Table 5.

**Table 5:** Results of the SEM for the research hypotheses.

Hypotheses	Paths	S.P.D	*t*	Result
*H_1a_*: Anxiety has a significant effect on shopping addiction	(Anxiety)➔(OSA)	0.16	2.09*	Accepted
*H_1b_*: Depression has a significant effect on online shopping addiction	(Depressi)➔(OSA)	0.18	2.29*	Accepted
*H_1c_*: Stress has a significant effect on online shopping addiction	(Stress)➔(OSA)	0.13	0.88	Rejected
*H_2_*: Self-control has a significant effect on shopping addiction	(Self_Con)➔(OSA)	0.14	0.99	Rejected
*H_3_*: Financial attitude has a significant effect on online shopping addiction	(Finan_At)➔(OSA)	-0.46	−9.11**	Accepted

** *p* < 0.01; * *p* < 0.05.

**Figure 3:**
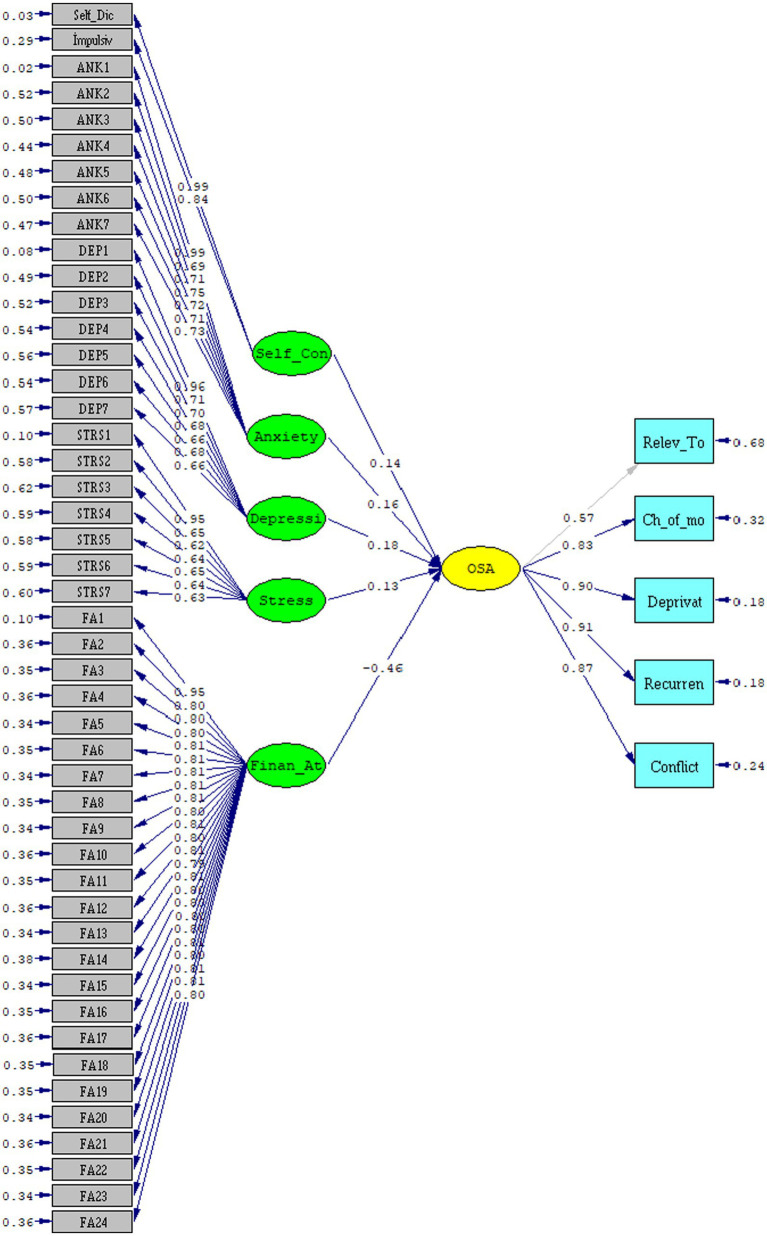
Results of the SEM for the research model. *χ*^2^ = 2340.62, df = 1,259, *p* = 0.000, RMSEA = 0.030.

The Chi-Square value (*χ*^2^) related to the model in Figure 3 was found to be *χ*^2^ = 2340.62, df = 1,259, *p* = 0.000, indicating significance at the 0.05 level. Examining the ratio of chi-square to degrees of freedom (*χ*^2^/df = 1.859), it was determined that it is lower than the acceptable threshold of 3, indicating an acceptable fit. Examination of the goodness-of-fit indices for the structural model yielded values of RMSEA = 0.030, RMR = 0.057, SRMR = 0.054, GFI = 0.91, AGFI = 0.91, CFI = 0.99, NFI = 0.99, and NNFI = 0.99. These values indicate that the structural model established here exhibits acceptable and excellent fit. The adequacy of the fit indices allows for the interpretation of path coefficients, and the results regarding the study hypotheses are presented in Table 5.

Examining the results related to the hypotheses of the study in Table 5.

It was determined that anxiety has a positively significant effect on online shopping addiction (*β*: 0.16, *t*: 2.09, *p* < 0.05). This result suggests that there will be a 0.16 increase in online shopping tendencies when there is a one-unit increase in participants’ anxiety levels.

It was determined that depression has a positively significant effect on online shopping addiction (*β*: 0.18, *t*: 2.29, *p* < 0.05). This result suggests that there will be a 0.18 increase in online shopping tendencies when there is a one-unit increase in participants’ depression levels.

It was determined that stress does not have a significant effect on online shopping addiction (*β*: 0.13, *t*: 0.88, *p* > 0.05).

The effect of self-control on online shopping addiction was found to be statistically nonsignificant (*β*: 0.14, *t*: 0.99, *p* > 0.05).

On the other hand, financial attitude was found to have a significant and negative effect on online shopping addiction (*β*: −0.46, *t*: −9.11, *p* < 0.01). This result suggests that, for every unit increase in participants’ financial attitude levels, there will be a decrease of 0.46 in online shopping tendencies.

## 4 Discussion

The results achieved in this study aiming to determine the predictive relationships between online shopping addiction and self-control, anxiety, depression, stress, and financial attitude variables are discussed in line with relevant literature. The literature review revealed a limited number of studies examining the predictability of online shopping addiction with self-control, anxiety, depression, stress, and financial attitude variables. Therefore, the findings of the research have also been discussed in comparison with the closest research findings.

In this study, it was determined that consumers exhibit moderate levels of financial attitude and self-control, with low levels of online shopping addiction.

In a study carried out by Müller et al. (35), approximately one-third of the participants were determined to be addicted to online shopping. Tang and Koh (36) found in their study that online shopping addiction is prevalent among university students.

The results achieved in a study carried out by Alkaya and Yağlı (37) indicated that the majority of students exhibit positive financial attitudes (37). In the study carried out by Yılmaz and Sevim (38), students’ financial behaviors were reported to be at a good level, with moderate levels of financial attitudes. Dayı and Esmer (39) determined in their study that academics are conscious of and exhibit balanced financial attitudes and behaviors.

In this study, it was determined that there is no significant relationship between participants’ levels of shopping addiction and their levels of self-control. In their study, Jiang et al. (40) reported that there is a negative relationship between self-control and online shopping addiction. Sümer and Büttner (41) identified self-control as a significant predictor of online shopping addiction in their study. Trotzke et al. (42) stated in their study that decreased self-control is a determining factor in shopping addiction. In a study carried out by Duong and Liaw (43), it was determined that internet experience has a significant negative impact on online shopping addiction, and the time spent on daily internet shopping and the frequency of daily internet shopping have a significant effect on online shopping addiction. In a study carried out by Ekşi et al. (44), it was found that self-control has a direct and high impact on social media addiction. Individuals with weak self-control tend to exhibit general procrastination behavior, leading to the development of social media addiction (44). The results reported in a study carried out by Korkman (45) indicated that self-control has a significant effect on anger, impulsivity, and risky behaviors. Nyrhinen (46) found in his study that low self-control facilitates online shopping addiction, leading to dissatisfaction with personal money management through indebtedness. Duroy et al. (47) expressed in their research that control and motivation loss are effective in online shopping addiction. Reviewing the literature, it was determined that there are studies with similar and different results in comparison to the present study. Different results are thought to originate from sample and cultural differences. The lack of a significant relationship between self-control and online shopping addiction levels in our study, attributed to the low levels of online shopping addiction among participants, suggests that different results may emerge with higher addiction levels and self-control. Additionally, the online nature of shopping may lead to differences compared to real-life shopping. In real-life shopping, individuals can notice how much they have bought, how much money is left in their wallet, how long they have been shopping, and whether they are tired, leading them to conclude their shopping. Similar external stoppers are absent in online shopping. Since individuals make purchases from the comfort of their seats, they may not realize the number of items purchased, the amount spent using a credit card, or the duration of time dedicated to shopping. In the absence of these factors, individuals may focus more on the pleasure of online shopping, potentially leading to increased shopping behavior. Therefore, the awareness levels of individuals in online shopping addiction should be considered.

Examining the results achieved in this study, it was determined that anxiety and depression have a positively significant impact on online shopping addiction, while stress does not have a significant effect on online shopping addiction. According to the research by Ayazoğlu et al. (17), some individuals experience changes in emotions during or after online shopping, such as regret, stress, anxiety, negative feelings, and excitement, as well as positive emotions like relaxation and distraction. In the same study, addictive factors were identified as impulses such as pleasure, relaxation, happiness, entertainment, affordable products, variety, comparison, and promotions; technological factors such as ease of use and usability; and psychological factors such as stress and boredom. In a study carried out by Aydın (48), a significant relationship was found between psychological symptoms such as anxiety and depression and shopping addiction. In the study carried out by Keskin and Günüç (15) aiming to examine why adults prefer online shopping and the factors leading to online shopping, the authors determined that usability, pleasure, stress, and depression affect online shopping addiction. According to the study carried out by Cojocariu et al. (49), there are seven variables thought to influence the potential development of online shopping addiction, and among them are negative emotions such as anxiety and stress. A study carried out by Müller et al. (35) demonstrated a significant relationship between anxiety, depression, and online shopping addiction. Given the results achieved in this study and those reported in the literature, it can be suggested that the levels of online shopping addiction will moderately improve when participants’ levels of anxiety and depression are ameliorated. As for the dimension of stress, the absence of a significant relationship between online shopping addiction in this study can be attributed to online factors influencing online consumer behaviors based on the structure of different regions and the variability of stress perception among individuals in different regions.

Given the results achieved in the present study, it was observed that financial attitude has a significant negative impact on online shopping addiction. In the study carried out by Ahmetoğulları (24), the use of digital banking, financial attitude, smartphone usage, social media usage, and pandemic anxiety was found to have a positive effect on online purchasing. Given the results reported by Kirezli (50), financial attitude also has an effect on online shopping. In this context, it can be argued that as participants’ levels of financial attitude improve, the levels of online shopping addiction will also decrease significantly.

## 5 Conclusion

Examining the content of the present study, it was observed that there is a lack of studies that collectively investigate the relationship between online shopping addiction, depression, anxiety, stress, and self-control and the financial attitude of consumers. The contribution of this study is that it fills a gap in the literature by identifying potential predictors of online shopping addiction. In this study, firstly, it was determined that consumers have moderate levels of financial attitude and self-control, and low levels of online shopping addiction. Secondly, based on the results of relationships and hypotheses among variables, it was found that anxiety, depression, and financial attitude are significant predictors of online shopping addiction, whereas stress and self-control do not significantly predict online shopping addiction. As a result, anxiety and depression were determined to positively influence online shopping addiction, whereas financial attitude was found to have a negative effect. To determine the reasons for online shopping addiction, it is recommended to conduct larger and more comprehensive studies with heterogeneous sample groups, take necessary precautions, increase awareness through education on online shopping addiction, and specifically focus on reducing individuals’ online shopping dependencies.

### 5.1 Ethical aspect of the research

The data collection process started with the submission of an ethical approval request to the Atatürk University’s Board of Social and Humanities Sciences Ethics Committee. The request was reviewed and approved (Session Nr. 21, Decree Nr. 321). Participants were provided with explanations regarding the purpose of the study, assurance of anonymous use of obtained data, and the voluntary nature of participation.

### 5.2 Limitations, future directions and implications

The strength of this study lies in the simultaneous examination of six variables, namely online shopping addiction, depression, anxiety, stress, self-control, and financial attitude, while other studies examined the effects of one or two variables. There are various limitations that need to be addressed in future studies. The results reported in this study indicate that participants have a low level of online shopping addiction. Future studies should investigate these effects on a sample consisting of individuals with moderate and high levels of online shopping to enhance the level of generalizability. Despite efforts to create a homogeneous sample, it should be noted that there are limitations in terms of generalizability. Moreover, the measurement of online shopping addiction, depression, anxiety, stress, self-control, and financial attitude variables is based on subjective judgments, and this limitation should be considered since participants may have provided biased responses. The fact that the survey instruments, including measurement tools, were administered online is also a limitation. It is thought that measurement errors could be minimized if surveys were conducted face-to-face.

This study, relying on a cross-sectional design, provides evidence of the relationship between variables. Longitudinal studies could be useful in determining the dynamics of the research model over time. The results reported in this study reinforce the understanding of online shopping addiction and provide important implications for practitioners. Future studies may include multiple-method assessments to comprehensively understand online shopping addiction. Moreover, in future studies, other variables not included in this study could be incorporated into the research process to identify variances that the models in this study could not explain.

## Data availability statement

The original contributions presented in the study are included in the article/supplementary material, further inquiries can be directed to the corresponding author.

## Author contributions

YE: Conceptualization, Data curation, Methodology, Resources, Validation, Writing – original draft, Writing – review & editing. GA: Investigation, Resources, Supervision, Validation, Writing – original draft, Writing – review & editing. BÇ: Data curation, Formal analysis, Methodology, Project administration, Visualization, Writing – original draft, Writing – review & editing. HT: Funding acquisition, Investigation, Software, Supervision, Writing – original draft, Writing – review & editing.
